# A Pilot Study of the Effect of Locomotor and Mechanical Loads on Elite Rowers During Competition Days

**DOI:** 10.3390/sports13080254

**Published:** 2025-08-01

**Authors:** Ferenc Ihász, Johanna Takács, Zoltán Alföldi, Lili Kósa, Robert Podstawski, Antonio Ferraz, Bożena Hinca, István Barthalos, Zsolt Bálint Katona

**Affiliations:** 1 Faculty of Health and Sport Sciences, Széchenyi István University, 9026 Győr, Hungary; ihasz.ferenc@sze.hu (F.I.); alfoldi.zoltan@ga.sze.hu (Z.A.); lili.kosa93@gmail.com (L.K.); barthalos.istvan@sze.hu (I.B.); 2 Department of Social Sciences, Faculty of Health Sciences, Semmelweis University,1085 Budapest, Hungary; takacs.johanna@semmelweis.hu; 3 Department of Physiotherapy, University of Warmia and Mazury in Olsztyn, 10-719 Olsztyn, Poland; podstawskirobert@gmail.com; 4 Department of Sport Sciences, Universidade de Beira Interior, 6201-001 Covilha, Portugal; antferraz@hotmail.com; 5 Department of Physical Education and Sport, University of Gdańsk, 80-854 Gdańsk, Poland; bozena.hinca@ug.edu.pl

**Keywords:** neuromuscular fatigue, performance, competitive rowing, explosive strength

## Abstract

(1) Background: Fatigue impacts neuromuscular performance, especially in endurance sports like rowing. The aim is to explore how continuous workload affects explosiveness and fatigue progression. This study examines acute fatigue during repeated race events by assessing vertical jump height, force output, and subjective fatigue over three consecutive days at the 2024 Hungarian National Rowing Championships. (2) Methods: Nine rowers (five women, four men; mean age 20.17 ± 1.73 years) competed in multiple 2000 m races over three days. Lower limb explosiveness was measured via countermovement jump (CMJ) using a Kistler force plate, pre- and post-race. Heart rate data were recorded with Polar Team Pro^®^. Subjective fatigue was assessed using the ‘Daily Wellness Questionnaire’. (3) Results: We found a significant difference in the pattern of the medians of the force exerted by males during the jump between the results of the Thursday preliminaries (ThuQ_Me_ = 13.3) and the second final (ThuF2_Me_ = −75.5). Women showed no notable changes. (4) Conclusion: Repeated high-intensity races induce neuromuscular fatigue in men, reflected in reduced explosiveness and increased subjective fatigue. Future research should incorporate biochemical markers to deepen the understanding of fatigue mechanisms.

## 1. Introduction

Physical activity is defined as any physical activity that results in a measured energy expenditure resulting from skeletal muscle contraction and a resting metabolic rate above 3.5 mL/kg/min or 1 metabolic equivalent (MET) [1]. Physical activity is classified according to intensity (low, medium, high) and the dynamic and static elements present in the movement. The adjective “dynamic” refers to endurance activities requiring the regular contraction of large muscle groups. It is characterized by the relative percentage of maximum aerobic power required to perform the activity, in other words, the maximum oxygen uptake [2]. Critical performance is the optimal performance achieved by using the athlete’s maximum physical and mental capacity. It can be defined by physical strength and endurance (aerobic and anaerobic capacity), technical skills (correct technique and movement competence), and mental effort (essential during physical tasks) [3,4,5]. Its main physiological determinant is oxygen, as this element is essential for most metabolic processes. During exercise, many different tissue systems need to increase their metabolism. Each tissue system has its own critical power, for example, the cardiac and respiratory systems, and different muscles or muscle groups [6]. Myocardium has the highest critical power relative to its maximum power because it has the shortest diffusion distance [7].

In sports with a high dynamic component, such as rowing, muscle fatigue is not a surprising factor. Muscle fatigue is a complex, multifaceted phenomenon and is most often defined as the inability of a muscle to maintain the desired level of force after prolonged use [8,9].

The task dependence of neuromuscular fatigue after different types of training has been widely studied, but little is known about the central and peripheral factors of fatigue after rowing. During rowing, approximately 70% of the total muscle mass is involved, as the upper and lower body muscles work in sync during the rowing motion. The quadriceps muscle plays a particularly important role in propelling the boat forward, as it presses against the footrest. In the neuromuscular function of the quadriceps muscle, the maximum voluntary torque was assessed as the root mean square (RMS·M^−1^) of the EMG signal during maximum voluntary contractions (MVC). After rowing 2000 m, a significant 20% decrease was observed in isometric and concentric maximum voluntary torque. The 2000 m rowing time trial caused significant quadriceps muscle fatigue. The data also showed that central factors contributed significantly to quadriceps muscle fatigue after rowing. Interestingly, previous studies have generally shown that peripheral mechanisms contribute less to quadriceps fatigue after short-term endurance training than central mechanisms [10].

Factors contributing to voluntary fatigue may be peripheral, central, or both. Separating the muscle and brain in separate studies can only provide a partial understanding of the mechanisms underlying the development of fatigue. Traditional ergonomic assessments to quantify muscle fatigue are limited to peripheral biomechanical, muscular, and physiological responses, i.e., they focus on the body. Fatigue is often described as a ubiquitous, multifactorial, and complex phenomenon that needs to be examined from a collective and tissue-specific perspective. It usually leads to a decline in performance. Related research defines it as a decrease in maximal voluntary effort. Fatigue or neuromuscular fatigue during training and competition affects body composition, metabolism, performance, and mental state. For instance, repeated maximal vertical jumps lead to progressive neuromuscular fatigue, manifesting as a significant reduction in jump height due to impaired force production and neural drive [11]. We can distinguish between two types of fatigue: physical and mental [12]. Physical fatigue is a condition resulting from intense training and/or competition, which can also lead to a decrease in performance due to changes in metabolism (decrease in resting metabolic rate and body fat). Mental fatigue is a psychobiological condition caused by prolonged cognitive activity. Athletes may experience a decline in concentration, motivation, and performance [13,14,15].

In recent studies, mental fatigue has also been shown to affect pacing strategies and decision-making processes during prolonged physical tasks, linking cognitive effort with biomechanical output [16]. Evidence from neuroimaging supports the involvement of the prefrontal cortex in regulating exertion perception, highlighting the role of brain regions in fatigue onset [17]. Additionally, hormonal responses such as increased cortisol and reduced testosterone during overreaching phases have been observed in relation to both mental and physical fatigue [18]. It is also known that chronic fatigue impairs mitochondrial function, disrupting energy metabolism and delaying recovery between training sessions [19]. Emerging wearable technologies now allow for the continuous monitoring of fatigue markers through heart rate variability, muscle oxygenation, and accelerometry, providing a more integrated view of fatigue in real-time performance contexts [20].

In this study we aimed to demonstrate that fatigue can develop in the body as the race day progresses in response to ongoing exertion and aimed to verify fatigue due to serial loading based on explosive exertion and subjective opinions.

## 2. Materials and Methods

**Figure 1:**
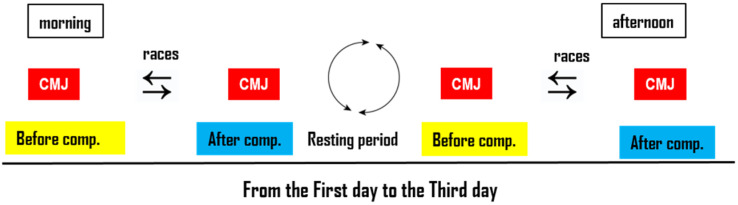
Flow chart of the research program. Notes: The figure shows the activities of the competitors during the three days of competition. On the morning before the race, after warming up, they performed a countermovement jump (CMJ), then boarded the boats and competed. Within 30 min after the competition, they performed another jump. This was followed by a rest period, the length of which depended on the competition schedule (1–3 h). These tasks were also performed in the afternoon.

The study was carried out at the “2024 Hungarian National Championships for Adult, Junior, Para, Amateur and Masters Rowing”, with a total of 12 competitors (21.25 ± 2.31 years old), seven women (22.41 ± 3.21), and five men (20.62 ± 1.64) at the Maty-éri course of the National Olympic Kayak-Canoe and Rowing Centre in Szeged, Hungary. The tests were conducted during the first three days of the four-day competition. During the three days, the competitors competed in preliminaries, medals, and finals in the boat classes given (Table 1, Figure 1).

**Figure 2:**
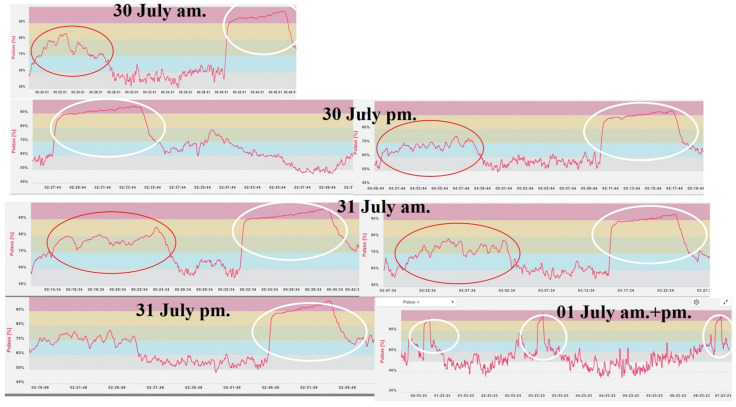
Heart rate pattern recorded for a competitor in all events. Notes: The total number of 2000 m races completed by each competitor over the three days. The white ellipse represents, heart rate pattern during a race, the red ellipse represents the warm-up before the race.

During the three days, the competitors were monitored before, during, and after the races using the Polar Team Pro^®^ system (Polar Electro, Kempele, Finland). The system consists of a chest strap containing a sensor device (Polar H7 Bluetooth 4.0 smart chest strap, Polar Electro, Kempele, Finland) with built-in ECG electrodes, integrated 10 Hz GPS, and 200 Hz microelectromechanical motion sensor (Polar Electro, Kempele, Finland). The data is transferred to the Polar Beat software (v3.5.4). The Polar Team Pro software (v3.5.4) records the heart rate (HR), calculates the time spent in different intensity zones (IZ_1–5_) (seconds), the covered distance (TD) (meters), summarizes the amount of exercise during the activity (TL), and estimates the energy expended (EC) (kcal) (https://support.polar.com/e_manuals/Team_Pro/Polar_Team_Pro_user_manual_English/introduction_to_polar_team_pro.htm accesed: 4 July 2024) (Figure 2).

The lower limb explosiveness during vertical jumps was measured on a force plateau, a Kistler type 9281A (Kistler Group, Winterthur, Switzerland), during a double-leg jump (CMJ). The MVJ v. 3.4 software package (“JBA” Zb. Staniak, Warsaw, Poland) was used for the measurement. The countermovement jump (CMJ) is one of the main tools used to assess the neuromuscular condition of elite athletes. Due to its reliability and validity, the CMJ test has become the gold standard test for monitoring neuromuscular fatigue in high-level sports [21]. Other authors also consider it useful due to its high repeatability and fatigue sensitivity, and it is currently the most appropriate test for detecting neuromuscular fatigue (NMF) [22]. Numerous experiments have been conducted using CMJ assessments to determine fatigue in the neuromuscular function of team athletes. In the scientific work of Gathercole et al. [22], intra-day and inter-day reliability comparisons showed high reliability, with no systematic changes in CMJ reproducibility. In professional rugby players, McLean et al. [23] used CMJ to monitor NMF and recommend that regular analysis of CMJ is a valuable tool for monitoring fatigue between seasons. Apart from jump height, the large number of variables showing acceptable reliability suggests that the CMJ strategy and performance remain stable over several days.

Each subject performed two vertical jumps, before and after his/her competition number, with maximum force on the force plate. The result of the jump after the competition was subtracted from the result of the jump before the competition. There was a 5 sec. break between CMJs. The jump with the higher lift was selected from the two trials. Before the jump, subjects performed a 5 min warm-up consisting of light exercises (e.g., running; arm, hip, and core curls; squats; followed by stretching exercises) and then a 2 min submaximal warm-up on a cycle ergometer (Monark 874 E, Sweden). They were instructed to pedal at 50–60 rpm and maintain a power of approximately 150 W. The questionnaire “Daily wellness questionnaire” included 4 questions on perceived energy level, quality of sleep, muscle fitness, and stress level. Each question was scored on a five-point scale, with 1 and 5 representing poor and very good well-being, respectively [24].

### Statistical Analysis

For descriptive analysis, means and standard deviations, as well as medians, were reported. Related-Samples Friedman’s Two-Way Analysis of Variance by Ranks was used to examine the differences in heart rate data over three days. Bonferroni correction was used to adjust the significance level for multiple comparisons to reduce the chance of making a Type I error during multiple comparisons. In this case, there were three pairwise comparisons; the adjusted significance level was 0.0167 (0.05/3). The level of significance was set at α = 0.05. Statistical analyses were conducted using IBM SPSS Statistics for Windows, Version 25.0 (IBM Corp. Released 2017, Armonk, NY, USA).

For descriptive analysis, means and standard deviations, as well as medians, were reported. Related-Samples Friedman’s Two-Way Analysis of Variance by Ranks was used to examine the differences in heart rate data over three days. During pairwise comparisons, significance values were adjusted using the Bonferroni correction for multiple tests. The level of significance was set at α = 0.05. Statistical analyses were conducted using IBM SPSS Statistics for Windows, Version 25.0 (IBM Corp. Released 2017, Armonk, NY, USA).

This research was approved by the István Széchenyi University Scientific Council (ETT) Committee on Scientific Ethics (SZE/ETT 5/2024 [V. 27.] DKH-2024/00043/2).

## 3. Results

Table 2 shows the physiological characteristics (HR_avg_, HR_max_, IZ1–5, TD, TL, RT, EC) recorded and calculated for all competitors during the three days of competition (using Polar Team pro telemetric devices). We have found significant differences between median heart rates (HR_avg_) χ^2^ = 6.222; *p* = 0.045, time spent in intensity zone 2 (IZ_2_) χ^2^ = 9.556; *p* = 0.008, and energy expenditure (EC) χ^2^ = 8.222; *p* = 0.016.

**Figure 3:**
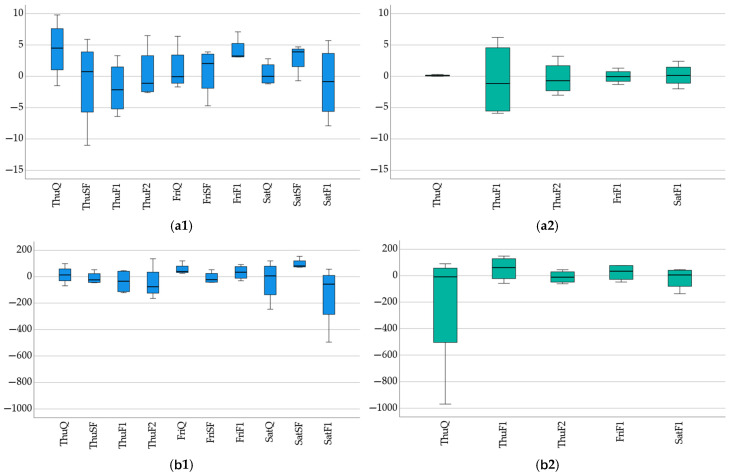

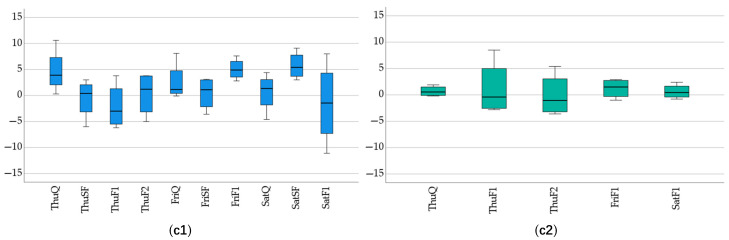
Difference between after and before measurements on height of jump (HJ) [cm] (**a**), maximal force delivered during jump (MF), [N] (**b**), and relative peak power (RMP), [W/kg] (**c**) among males (**a1**,**b1**,**c1**) and females (**a2**,**b2**,**c2**). Notes: Thu: Thursday, Fri: Friday, Sat: Saturday, Q: qualifying heat, SF: semi-final, F1: final 1, F2: final 2. Y-axis: zero means there was no difference between before and after measurements, negative values = after > before, positive values = before > after.

**Figure 4:**
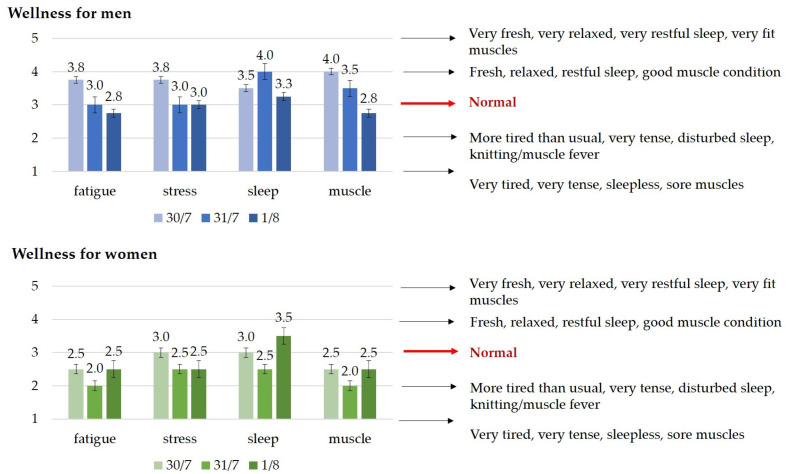
‘Daily wellness questionnaire’ results (mean ± std error) for the three competition days, each morning. Notes: The “Daily Well-Being Questionnaire” contained four questions about perceived energy levels, sleep quality, muscle condition, and stress levels. Each question was rated on a 5-point scale, where 1 meant poor and 5 meant very good well-being. The scores for the three days are represented by three different colors.

On the first day of the competition, the men got into the boats four times and completed the 2000 m distance four times, while the women competed three times over the same distance. After and before the first race, the difference in jump height was in the revisedpositive range, with ThuQ_Me_ = 4.5. This was also true for the second event, with ThuSF_Me_ = 0.8, except that the first quartile (Q1) value was −8.4. The first ThuF1_Me_ = −2.2 and the second final median value, ThuF2_Me_ = −1.1, were in the negative range (Figure 3(a1). The medians of the men’s jumps on Friday and Saturday were in the positive range, except for the Saturday final, SatF1_Me_ = −0.9. We found no significant difference between the women’s jump performances on Thursday, Friday, and Saturday, although the medians of the two finals on Thursday were in the negative range. The median of the second final was ThuF1_Me_ = −1.15, and the first quartile Q1 = −5.73 was significantly in the negative range (Figure 3(b1)). We found a significant difference in the pattern of the medians of the force exerted by men during the jump between the results of the Thursday preliminaries (ThuQ_Me_ = 13.3) and the second final (ThuF2_Me_ = −75.5) (Figure 3(a2)). In the case of women, we found a significant shift into the negative range in the first competition, ThuQ_Me_ = −8.50, and the first quartile, Q1 = −736.75, as well as a difference between the Thursday semifinals and the final, ThuF2_Me_ = −11.0 (Figure 3(b2)). The height of the jump (HJ) and the relative peak power (RMP) patterns reported in between are consistent with minor differences independent of gender (Figure 4).

The men’s responses on the first day were close to four in all four categories, which means that “fresh, restful, calm sleep, good muscle condition” was characteristic. A significant deterioration can be observed on the third day, where muscle quality deteriorated, which corresponds to fatigue. The women’s responses were slightly below “normal” in all categories on the first day, but on the second and third days, they dropped to around 2 in all categories, which can be described as “more tired and usual, very tense, disturbed sleep, knotted muscle/fever.”

## 4. Discussion

This study examined acute fatigue in mechanical properties and neuromuscular activity over three consecutive days of the 2024 Hungarian National Rowing Championships. Using CMJ (Countermovement Jump) assessments, we analyzed variations in jump strength to evaluate fatigue progression. In the men’s group, a significant decrease in jump height and force output was observed after the final race on the first day compared to the first race. However, no notable differences in acute fatigue were found among female rowers.

Subjective feedback further supported these findings, revealing significant increases in fatigue scores between the first and third days in male athletes. Additionally, sleep quality declined between the second and third days, and muscle fatigue worsened between the first and third days. These trends suggest cumulative fatigue effects over consecutive race days, particularly in male rowers.

Josh and colleagues [25] investigated the dynamics of sleep, somatic/psychological experiences, and physical performance before, during, and after the Tour de France (TDF). Objective and subjective sleep data, self-reported perceived experience, and objective physical performance data were collected daily from eight elite male cyclists over a 6-week period, including the 3-week TDF and the 11-day pre- and post-race periods. Changes in sleep, perceived exertion, and objective training load were observed before, during, and after the Tour de France. The simultaneous decrease in muscle soreness and potentially significant improvement in subjective sleep quality in the post-race period may indicate that increased psychophysiological load contributed to sleep disturbances during the race. Several previous studies have shown that athletes in different sports report sleep durations within an appropriate range, but subjective assessments of sleep quality deteriorated and the time needed to fall asleep increased, particularly as the competition days progressed [26,27].

Fatigue studies have traditionally been conducted in controlled laboratory environments using models such as dynamic knee extensions, sustained contractions, or sprint tests on cycle ergometers. While valuable, these methods may not fully replicate the demands of competitive sports. To address this gap, our study assessed rowers under real competition conditions. Similar findings have been reported in other high-intensity sports, such as handball, where repetitive sprints, jumps, and directional changes lead to significant performance degradation [28]. Research on elite Danish handball players has also demonstrated reductions in maximal voluntary contraction (MVC) and rapid force development (RFD), particularly in leg muscles, during matches.

The underlying causes of fatigue may include both central and peripheral mechanisms. Peripheral fatigue is often associated with metabolic factors such as glycogen depletion and intracellular pH reduction [29].

The underlying causes of fatigue can include both central and peripheral mechanisms. Peripheral fatigue is often associated with metabolic factors such as glycogen depletion and a decrease in intracellular pH [30]. Glycogen depletion and calcium ion (Ca ^++^) flux regulation, as well as disturbances in these processes in the cell–matrix, can be linked to fatigue occurring during moderate-intensity, prolonged activity. It is important to note that these are not necessarily the only factors contributing to the development of fatigue. During sustained activity, a decrease in mitochondrial function contributes to fatigue—studies supporting and refuting this hypothesis have been published [31]. Our research was based on subjective data. Based on these, the responses to the Wellness questionnaire indirectly confirm the above. When compared with the content of Table 1, which shows the competition load, the subjective assessment of sleep and muscle fatigue is consistent with this. Peripheral fatigue results from a decrease in muscle function caused by overload originating from non-central nervous system mechanisms. A common symptom of fatigue is a feeling of exhaustion that occurs because of overload, such as intense or prolonged physical activity. These conditions can increase fatigue to a level that can compromise health. Although the etiological aspects of peripheral fatigue are often associated with limitations of the regulatory system (neurological, endocrine, immunological, and muscular), the final mediation occurs in the muscle cells. The test we conducted has several limitations, but it still provides information to the team’s professional leaders about the current condition of the competitors, whether it is necessary to recognize exhaustion, or make decisions about the likelihood of injury. However, our study did not include direct biochemical measurements to confirm these metabolic changes.

### 4.1. Recommendations

Our findings highlight the need for further research into the biochemical and metabolic aspects of fatigue in endurance sports. Future studies should integrate enzymatic and metabolic analyses, such as muscle glycogen depletion and lactate accumulation, to directly confirm physiological fatigue markers.

Extending assessments to include the fourth day of competition could provide a more comprehensive picture of cumulative fatigue progression. Since subjective fatigue scores aligned with objective performance reductions, incorporating self-reported fatigue metrics alongside physiological assessments could enhance real-world fatigue monitoring.

Additionally, investigating targeted recovery strategies—such as optimized nutrition, hydration, and neuromuscular recovery techniques—could provide valuable insights into mitigating fatigue and improving performance sustainability for endurance athletes.

### 4.2. Limitations and Strengths

It is possible that the final day would have shown even stronger fatigue effects. Furthermore, while reductions in explosive power are a known consequence of neuromuscular fatigue, our study could only infer this relationship indirectly. Future research incorporating direct neuromuscular activity measurements, such as electromyography (EMG), could provide more definitive evidence. It would have been advantageous to assess the heart rate variability (HRV) of the participants immediately upon awakening, prior to the competition, and during rest periods. The Polar Team Pro software would have been well-suited for this purpose.

Although heart rate changes were monitored, they did not significantly correlate with acute fatigue levels, suggesting that heart rate may not be a reliable standalone marker for neuromuscular fatigue in this context. Despite these limitations, a key strength of this study is its real-world competition setting, allowing for a more ecologically valid assessment of fatigue compared to traditional laboratory studies. By evaluating fatigue progression in competitive rowers, our research contributes valuable insights into how sustained high-intensity exertion impacts athletic performance.

## 5. Conclusions

Our findings suggest that repeated high-intensity race events induce acute neuromuscular fatigue, particularly in male rowers, as evidenced by declines in jump height and force output. Subjective fatigue scores further support this conclusion, highlighting the physiological strain imposed by consecutive race days.

While our study provides valuable insights into fatigue responses in real-world competition settings, further research is needed to explore underlying biochemical mechanisms. Incorporating metabolic and enzymatic analyses could provide a clearer understanding of fatigue progression and its physiological effects on performance.

## Figures and Tables

**Table 1:** Races of the competitors included in the study, over three days of competition.

	First Day		Second Day		Third Day		Total
Sex	Morning	Afternoon	Morning	Afternoon	Morning	Afternoon	
2	ns8+	ns8+		ns2-		ni8+	4
2	ns8+	ns8+		ni4-		ni8+	4
2	ns8+/ns4x	ns8+/ns4x		ni4-		ni8+	6
1	fs4x/fs8+	fs4x/fs8+	fs1x	fs1x	fs4+	fs2x (2)	9
1	fs4x/fs8+	fs4x/fs8+	fs1x	fs1x	fs4+	fs2x (2)	9
2	ns8+	ns8+	nf2-	ni4-/nf2-	ni8+		6
2	ns8+	ns8+	nf2-	ni4-/nf2-	ni8+		6
1	fs4x/fs8+	fs4x/fs8+	fs2-	fs2-	fs4+	fs2x (2)	9
1	fs4x/fs8+	fs4x/fs8+	fs2-	fs2-	fs4+	fs2x (2)	9

Notes: ns2- = women’s adolescent pair-oar, ns4x = women’s adolescent quadruple, ns8+ = women’s adolescent coxed eights, ni4- = women’s junior quadruple, ni8+ = women’s junior coxed eights, nf2- = women’s adult pair-oar, fs1x = male adolescent skiff, fs2- = male adolescent pair-oar, fs2x(2) = male adolescent coxless pair-oar (two runs), fs4+ = male adolescent coxed quadruple, fs4x = male adolescent coxless quadruple, fs8+ = male adolescent coxless quadruple.

**Table 2:** Comparison of physiological characteristics during the three days of competition (N = 9).

	Mean Rank			χ^2^	*p*	Post Hoc	Day 1			Day 2			Day 3		
	Day 1	Day 2	Day 3				M	SD	Mdn	M	SD	Mdn	M	SD	Mdn
HR_avg_	2.4	2.2	1.3	6.222	0.045	1 > 3, 1 = 2, 2 = 3	130.1	4.2	128.5	130.1	4.2	129.0	130.1	4.2	116.0
HR_max_	1.8	2.2	2.0	0.941	0.625		194.6	5.6	196.0	197.6	5.8	198.0	197.9	9.3	198.0
IZ_1_	2.2	1.7	2.1	1.556	0.459		0:56:18	0:07:22	0:55:55	0:41:48	0:16:26	0:39:46	1:16:56	0:54:13	0:49:33
IZ_2_	2.8	1.3	1.9	9.556	0.008	1 > 2, 1 = 3, 2 = 3	0:47:28	0:11:53	0:52:55	0:33:05	0:09:47	0:31:08	0:46:01	0:39:24	0:32:41
IZ_3_	2.4	2.1	1.4	4.667	0.097		0:18:29	0:06:53	0:17:15	0:13:43	0:07:40	0:14:47	0:10:31	0:10:32	0:06:53
IZ_4_	2.1	1.9	2.0	0.222	0.895		0:08:37	0:05:41	0:05:55	0:07:14	0:04:25	0:06:16	0:08:17	0:03:42	0:06:44
IZ_5_	2.4	1.8	1.8	2.667	0.264		0:07:24	0:04:00	0:08:02	0:06:14	0:03:43	0:07:04	0:06:35	0:05:04	0:06:55
TD	2.2	1.6	2.2	2.667	0.264		2575.2	635.2	2441.0	2121.6	851.5	2001.0	3752.6	2705.6	2176.0
TL	2.4	2.0	1.6	3.556	0.169		114.6	23.3	110.5	89.4	37.1	74.0	96.9	83.3	61.0
RT	2.4	1.8	1.8	2.667	0.264		15.3	6.8	13.4	11.2	6.0	10.0	15.9	18.6	8.5
EC	2.3	1.2	2.4	8.222	0.016	1 = 3 > 2	1151.9	87.9	1126.5	934.3	238.0	892.0	1587.0	843.6	1078.0

Notes: HR_avg_: average heart rate [beat × min^−1^], HR_max_: maximum heart rate [beat × min^−1^], IZ_1–5_: intensity zones (1–5), (where zone 1 represents 50–60% of the maximum heart rate and zone 5 represents 100% of the maximum heart rate), TD: total distance [m], TL: training load, RT: recovery time [hour], EC: energy consumption [Kcal].

## Data Availability

The data presented in this study are available upon request from the corresponding author. The data are not publicly available because they belong to minors.
